# Biomedicine Innovations and Its Nanohydrogel Classifications

**DOI:** 10.3390/pharmaceutics14122839

**Published:** 2022-12-18

**Authors:** Sifiso S. Makhathini, Sipho Mdanda, Pariksha J. Kondiah, Moosa E. Kharodia, Karl Rumbold, Imhotep Alagidede, Yashwant Pathak, Zain Bulbulia, Thankhoe A. Rants’o, Pierre P. D. Kondiah

**Affiliations:** 1 Department of Pharmacy and Pharmacology, School of Therapeutic Sciences, Faculty of Health Sciences, University of the Witwatersrand, Johannesburg, 7 York Road, Parktown, Johannesburg 2193, South Africa; 2 FH Campus Wien, University of Applied Sciences, Vienna, Höchstädtpl. 6, 1200 Wien, Austria; 3 Simon Diedong Dombo University of Business and Integrated Development Studies, Bamahu Box WA64 Wa, Upper West Region, Ghana; 4 Wits Business School, University of the Witwatersrand, 2 St Davids Pl &, St Andrew Rd, Parktown, Johannesburg 2193, South Africa; 5 USF Health Taneja College of Pharmacy, University of South Florida, 12901 Bruce B Downs Blvd, MDC 030, Tampa, FL 33612-4749, USA; 6 Faculty of Pharmacy, Airlangga University, Surabaya 60115, East Java, Indonesia; 7 Policy Research & Advisory Services Branch, Gauteng Office of Premier, 1 Central Place 30 Rahima Moosa Street Newtown, Johannesburg 2113, South Africa; 8 Pearson College London Alumni (Pearson plc), London WC1V 7BH, UK

**Keywords:** Nanogel, polymeric, crosslinking, nano-delivery, active targeting, mechanisms of drug release, nanotechnology, multifunctional nanogels

## Abstract

As one of the most cutting-edge and promising polymer crosslinked network nanoparticle systems. Polymer nano-sized hydrogels (nanogels) have been a hot topic in the biomedical field over the last few decades. Due to their unique characteristics, which include their relatively high drug encapsulation efficiency, ease of preparation, high tunability, low toxicity, high stability in serum and responsive behavior to a range of stimuli to facilitate drug release. Nanogels are thought to be the next generation of drug delivery systems that can completely change the way that drug delivery systems have an impact on patients’ lives. Nanogels have demonstrated significant potential in a variety of fields, including chemotherapy, diagnosis, organ targeting, and delivery of bioactive molecules of different dimensions. However, the lack of substantial clinical data from nanogels becomes one of the major barriers to translating the nanogel concept into a practical therapeutic application for many disease conditions. In addition, nanogel safety profiles have been the major concern that hinders it advancement to the clinical trial phase. This review aims to emphasize the unique properties of nanogels as delivery systems for a variety of bioactive molecules over other nano-delivery systems. Also, this review attempts to give insight into the recent progress in nanogels as a carrier in the field of nanomedicine to overcome complex biological barriers. Relevant scientific data and clinical rationale for the development and the potential use of nanogel as a carrier for targeted therapeutic interventions are discussed. Finally, the concluding points of this review highlight the importance of understanding the long-term toxicity profile of nanogel within the biological system to fully understand their biocompatibility.

## 1. Introduction

Over the years, multidisciplinary studies have demonstrated the role of nanomedicines and nano-based drug delivery systems as an alternative therapeutic strategy in the management, prevention, diagnosis, and treatment of diseases [1,2,3]. Parameters, including the route of administration, safety profile of the material, and drugs’ pharmacodynamic, pharmacokinetic, and physiochemical properties, have been used to identify suitable nanocarriers in order to achieve the desired therapeutic or diagnostic outcomes [4]. The development of nanocarriers with these characteristics have been shown to improve the drug’s therapeutic efficacy and minimize potential side effects of the treatment by exploiting the pathophysiology of the diseased site. While it is unlikely to find or design an ideal nano-delivery system that meets all therapeutic demands, designing a multi-functional system with appropriate pharmacokinetic, biocompatibility, and microenvironmental responsiveness is highly encouraged. Polymer-based drug delivery systems (DDS) are among the DDS with adjustable properties that have gained considerable attention in the field of nanomedicine, pharmaceutics and bio-nanotechnology in the fight against disease [5]. Polymeric DDS with controlled therapeutic release and cell targeting, in particular, offer the potential to treat a variety of diseases, including malignancies and other complex disorders, while reducing drug side effects [6]. Nanogels are one of the most promising polymeric DDSs that have been investigated, and they have become the topic of various studies focusing on new formulation strategies and applications of novel nanocarriers with superior therapeutic effects [2,7,8,9].

Nanogels are commonly defined as nanohydrogels with a three-dimensional nanosized structure formed by chemically or physically cross-linked swellable polymer network systems that can retain large quantities of water while maintaining their structural integrity [7,8]. They have a well-defined spherical structure, and with recent advancements in their fabrication processes, nanogels of various shapes can be produced [9]. Nanogels are also known for their capacity to accommodate a wide range of guest molecules, ranging from inorganic nanoparticles to biomacromolecules like proteins without compromising their gel-like properties. Various types of polymer networks can be used to form nanogels, including natural and synthetic polymer networks or a combination of both. In particular, polymers containing polar functional groups such as –OH, –CO_2_H, –NH_2_, –CONH_2_, –CONH, and –SO_2_H are common in the synthesis of nanogels [10,11]. These polymer chemical compositions can be altered to improve the benefits of nanogels as drug delivery systems to achieve the intended biomedical application. Chemical modification of the polymer forming the network system can improve the active targeting ability of a delivery system to achieve high drug localization at the disease site. In recent years nanogel-based DDSs have gained acceptance from the scientific community as the new and promising alternative strategy that has the potential to change the therapeutic landscape of nanomedicine and improves lives.

Despite being a hot topic in multidisciplinary domains with diversity in their applications, nanogels have scarcely been adopted for clinical practice. This is due to numerous safety-related issues that must be overcome. However, nanogels that are in the market have shown some excellent therapeutic outcomes, include Oxalgin nanogel loaded with a non-steroidal anti-inflammatory drug, diclofenac, in menthol nanogel for application in analgesia and inflammatory conditions. Zyflex nanogel is another example made from muscle relaxant, thiocolchicoside, in menthol nanogel for use in muscle spasms [1]. Voriconazole loaded in gold–silver cuprous oxide composite nanogels is currently being assessed in clinical trials for the treatment of severe drug-resistant bacterial keratitis [12]. Although the progression of nanogels into different stages of clinical trials is very slow, the results published suggest that more nanogel formulations may reach different levels of clinical trials in the near future.

In this review, we provide a summary of different preparation methods, compositions, and properties of nanogels leading to their targeting and stimuli-responsive ability, 3D printing of nanogels, biomedical applications with a focus on chemotherapy, and finally, toxicity-related issues will also be discussed. The features to be discussed form the bases of the advancement of nanotechnology and the usefulness of nanogel as a delivery vehicle. In addition, the advancement of nanotechnology regarding nanogel supported by the latest references from the literature and other unique properties regarding nanogels will be discussed in this review. 

## 2. Different Synthetic Approaches to the Design of Nanogels

**Figure 1:**
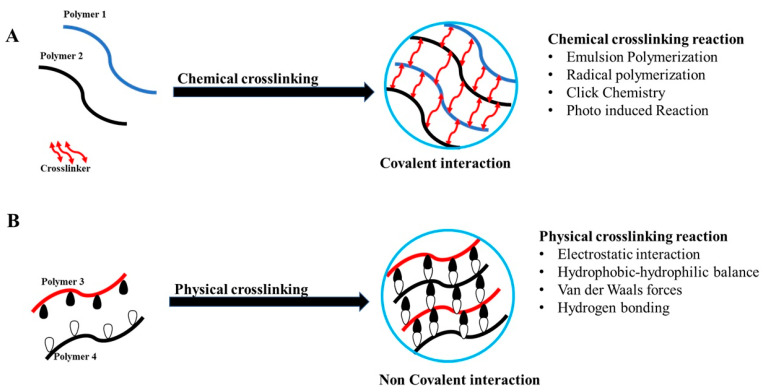
The design of nanogels (NG) using chemical and physical crosslinking processes is depicted. (**A**) Covalent bond formation (crosslinking points, in red) using different chemical reactions generated by the interaction of reactive moieties between polymers 1 and 2. (**B**) The self-assembly of a nano-scaffold is enabled by the physical interactions between polymers 3 and 4 [2].

Preparation techniques for nanogel are based on their cross-linking structure and the polymer building blocks making the nanogels system, which are commonly classified into physically crosslinked and chemically crosslinked self-assembled nanogels. While the chemically cross-linked nanogels result from covalent interactions forming a stable system, physically cross-linked nanogels are formed by reversible/non-covalent interactions. (see Figure 1) [13,14]. As a result of diverse nanogel applications in the biomedical field, a lot of research has been focused on simplifying and optimizing the synthetic methods of nanogels [5,15]. The following sections summarise some of the common nanogel preparation methods and their examples.

### 2.1. Physical Crosslinking 

**Scheme 1:**
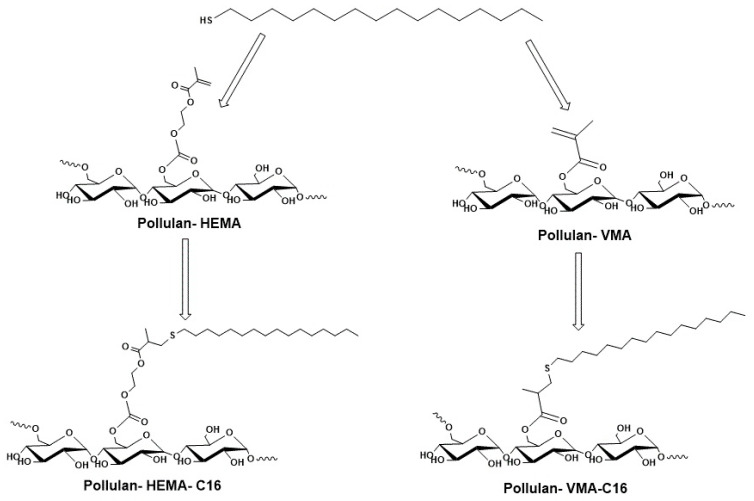
Synthesis of pullulan-C16.

Physically crosslinked nanogels, also known as pseudo gel/ supramolecular particles, are formed by weaker interactions between the polymer reactive groups in an aqueous medium under mild conditions. These interactions, including van der Waals forces, hydrogen bonding, and electrostatic interactions, can initiate the self-assembly of polymer material into nanogels depending on the functionalities grafted onto the polymer backbone (see Figure 1B) [16,17]. Amphiphilic block copolymers and the complexation of oppositely charged polymeric chains have been extensively used in the synthesis of nanogels [18]. In addition to the ease of preparation of nanogel using this technique, the physical crosslinking method does not require the use of toxic cross-linkers or catalysts, and no side-products are formed during the nanogel preparation process [19]. Although physically cross-linked nanogels are relatively less stable than chemically cross-linked nanogels (see Section 2.2), the reported instability of nanogel in biological media can be overcome by modifying the properties of polymer material to gain more stability. For instance, cholesterol-modified pullulan (CHP) is the earliest example of amphiphilic polymeric material used to form physically cross-linked nanogels, especially for protein delivery [20]. CHP-based nanogels for protein delivery were found to have low bloodstream stability, resulting in premature drug release. Synthesis of nanogels from polysaccharides functionalized with larger hydrophobic side chains was one of the interventions proposed to address the serum instability issue [21]. Polysaccharides attached with short poly(N-isopropyl acrylamide) (PNIPAM) chains are typical examples of physically cross-linked thermoresponsive nanogels formed by the hydrophobic nanodomains generated by dehydration of PNIPAM [20]. In one of the recent examples, Ferreira and colleagues synthesised and characterized self-assembled nanogels made of hydrophobized pullulan. They first grated pullulan with hydroxyethyl methacrylate or vinyl methacrylate as a linker followed by the attachment of hydrophobic 1-hexadecanethiol to form an amphiphile that can self-assemble in water via the hydrophobic interaction among alkyl chains (see Scheme 1). After the characterization of the physiochemical properties of nanogels, their results indicated that the modification of pullulan with hydroxyethyl methacrylate with fewer hydrophobic chains grafted yielded a more stable system [22].

### 2.2. Chemical Crosslinking

The most common and multipurpose technique for preparing highly stabilized nanogel is via chemical cross-linking (covalent interactions). Different chemical cross-linking strategies that have been reported in the literature in recent years will be discussed briefly and with relevant examples in the following sections.

#### 2.2.1. Inverse Emulsion Polymerization (IEP)

The IEP method has been used to produce kinetically stable monodisperse solutions of emulsion (water-in-oil [W/O]) with the desired size. Reviews have been published summarizing recent works reporting on nanogel synthesis using the IEP technique [7]. This method involves the emulsification of the polymer/monomer precursor solution in a continuous organic medium in the presence of oil-soluble surfactant and cross-linking agents [23,24]. Several examples using this approach have continued to surface, highlighting its applicability in the construction of kinetically stable monodispersed particles, particularly stimuli-responsive nanogels. In 2019, Hajebi, Sakineh, et al. prepared a novel dual temperature- and pH-responsive Dox-loaded hybrid and hollow nanogel to investigate their Dox release rate at different pH values (3.5 and 7.5) [25]. In another study reported by Gao, Feng, et al., biodegradable temperature and redox-responsive hybrid nanogels [Poly(N-vinylcaprolactam)/ Fe_3_O_4_ (NCGs)] with magnetic properties were successfully synthesized using the IEP technique with good structural integrity in the presence of N, N’-bis(acryloyl) cystamine (BAC) as the cross-linking agent [26]. Both studies highlighted above indicate that the IEP method could be a convenient method for the efficient synthesis of nanogels from a range of biomaterials. In addition, using covalent cross-linking agents within inverse nanoemulsion or nanogels can aid in the entrapment and prevention of leakage of cargo during redispersion in an aqueous medium.

#### 2.2.2. Reversible Addition–Fragmentation Chain Transfer (RAFT) Polymerization

**Scheme 2:**
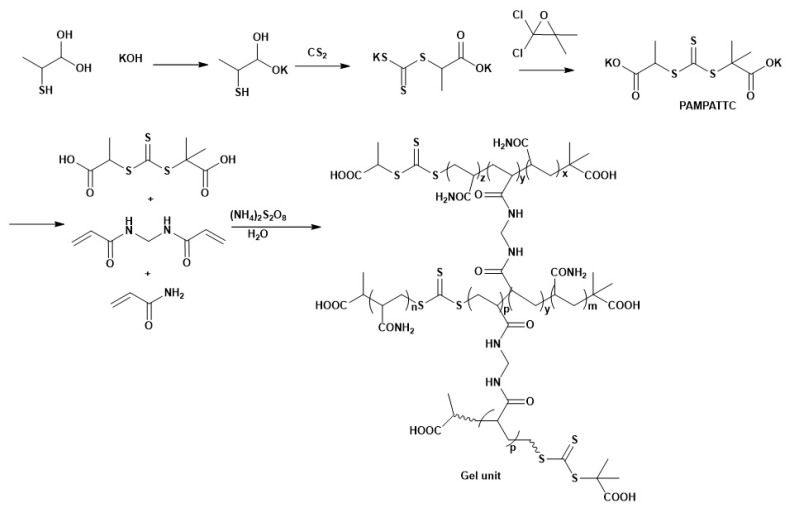
Synthesis process of hydrosoluble reversible addition–fragmentation chain transfer (RAFT) agent for the preparation of polyacrylamide gel dispersions [29].

The reversible addition–fragmentation chain transfer (RAFT) process is the other most-used polymerization technique for engineering complex polymeric nanostructures for drug delivery, and it is common in the synthesis of nanogels. For example, Photo-RAFT-mediated polymerization-induced self-assembly (photo-RAFTPISA) of poly(N-acryloyl glycinamide) (PNAGA) using N,N′-methylene bis(acrylamide) as a crosslinker and poly(oligo(ethylene glycol) methyl ether acrylate) as both a stabilizer and a macromolecular chain transfer agent were used to prepare thermosensitive nanogels. Under UV irradiation and low temperatures (3 °C), very stable, spherical nanogels with narrow polydispersity were generated within one hour [27]. Yang, Ziteng, et al. also reported another study using the same preparation method within the same year. They prepared micro/nano-polyacrylamide gel dispersions via one-pot RAFT polymerization in the presence of pre-synthesized S-(2-propionic acid)-S′-(2-methylpropionic acid) trithiocarbonate (PAMPATTC) as a RAFT agent with success (see Scheme 2). Studies highlighted above and other referenced material suggest that the RAFT technique is an efficient strategy to obtain a well-defined nanogels system in aqueous dispersion. In addition, these studies also revealed that a good balance between fragmentation and reinitiation is crucial in achieving a controlled polymerized chain structure [27,28,29,30]. In comparison to other radical polymerization techniques, RAFT has demonstrated versatility in application, since it can be also used for most monomers polymerizable under relatively mild conditions [28].

#### 2.2.3. Click Chemistry Crosslinking Polymerization

**Figure 2:**
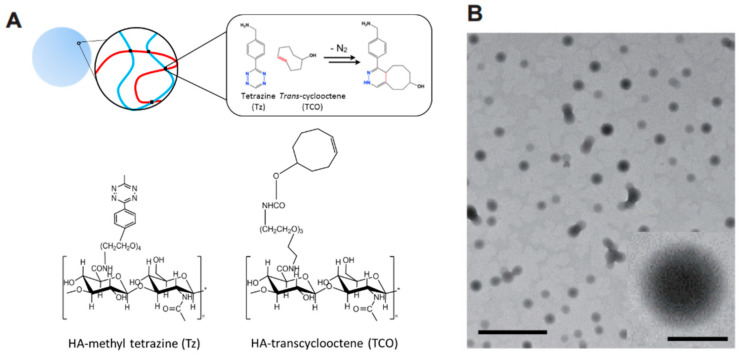
(**A**) Schematic representation of HA nanogels crosslinked by methyl tetrazine (Tz) and trans-cyclooctene via click chemistry (TCO). (**B**) HA nanogel Cryo-TEM picture (scale bar = 500 nm) [34].

Click chemistry is the most instrumental approach in creating materials with highly controlled structures in high yields under mild conditions for a short period of time. It has been extensively explored in nearly every field of modern chemistry, from drug discovery to materials science [31]. This approach involves the use of biomaterials containing azide and alkyne groups to form a stable conjugate. Copper-catalyzed azide-alkyne cycloaddition (CuAAC) click reaction, copper-free click reaction, and pseudo-click chemistry are a few typical examples of click reactions used for particle functionalization [32]. In drug delivery, it is one of the research topics, among others, in which these synthetic approaches have found applicability. For example, Phan, Quoc Thang, et al. developed zwitterionic redox-responsive DOX-loaded nanogels prepared via a simple one-pot “click” reaction of α, ω-functionalized poly(sulfobetaine)s (FPSBs) and cystamine. The FPSBs were formed without by-product formation via ATRP polymerization in the presence of Bromo-2-methyl-propanoate containing furan-maleimide adducts as an initiator. This was followed by the conjugation of alkyne-functionalized thiolactones onto PSBs through an azide-alkyne “click” reaction (AAc). The author further detailed the mechanism of aminolysis reaction to form a highly reactive thiol group using cystamine crosslinkers, resulting in the formation of nanogel networks under UV-irradiation conditions [33]. Hyaluronate nanogels crosslinked via click reaction between methyltetrazine (Tz) and trans-cyclooctene (TCO) for topical application is another recent example employing this technique reported by Choi, Hyunsik, et al. (see Figure 2). Drugs were encapsulated and delivered noninvasively via transdermal and sublingual routes using HA nanogels prepared using click chemistry [34]. In addition, some works using this technique have highlighted that the lack of reactive groups for some of the starting materials is one of the limitations associated with using this method. However, grafting azide and the triple bond on the backbone of the polymer can help overcome this limitation [35].

#### 2.2.4. Photo-Induced Crosslinking Polymerization

Alternatively, photochemistry is another crosslinking technique used to form functionalized stable polymer assemblies via either polymerizable or dimerizable units upon exposure to specific photo irradiation [36]. Similar to physical cross-linking, this technique does not require cross-linking agents or catalysts and purification steps before and after the crosslinking process [15,37]. Photoresponsive nanogels using a double hydrophilic block copolymer containing coumarin that dimerizes when treated with UV light > 310 nm using this method were reported. The initial steps involve the formation of micellar aggregates from water-soluble diblock copolymer at temperatures above its lower critical solution temperature (LCST), and subsequently photo-cross-linked as a result of the dimerization of coumarin moieties initiated upon UV irradiation at λ > 310 nm. Upon cooling to T < LCST, the water-soluble nanogels were successfully generated [38,39]. In another example, recently, a UV-induced nanonetwork preparation method was used to construct gelatin methacrylate NGs with pH-dependent properties. The subsequent system was used as a DDS for the transdermal distribution of hydrophilic macromolecules [40]. Despite the high efficiency of photo-induced crosslinking, several studies have reported that the initiator could potentially cause high cytotoxicity levels in the gels generated. Therefore, selecting a suitable photoinitiator is critical to the preparation of biocompatible gel networks using this method [41].

#### 2.2.5. Disulfide Based Crosslinking

Covalent disulfide bond (S-S) linkages are formed by the oxidation of sulfhydryls (SH) groups found in natural peptides and proteins [5,15]. These bonds have been widely used in drug discovery and polymer designs, and they offer a simple route for developing biodegradable cross-linked drug delivery systems due to their reversibility and relative stability in plasma [42,43]. The high redox potential that exists within the biological system between the oxidizing extracellular environment and the intracellular reducing environment makes the disulfide bond linkages a perfect tool for designing DDS with redox-responsive properties. For example, the disulfide links demonstrate stability in extracellular medium while they undergo cleavage upon exposure to glutathione antioxidant produced within the cells. In drug delivery, this concept has been used in the design of nanosystems capable of intracellular drug delivery. Also, biodegradable nanogels of this nature were developed from this concept. Disulfide-functionalized dimethacrylate crosslinkers were used in the preparation of nanogels reported by Matyjaszewski et al. Their findings demonstrated that in the presence of glutathione, nanogels degraded into individual non-toxic polymeric chains/residue facilitating the release of encapsulated bioactive substance [44,45]. Redox-sensitive doxorubicin-loaded nanogel is another example of nanogels constructed using amphiphilic copolymer containing hydrophilic oligoethyleneglycol (OEG) and hydrophobic pyridyldisulfide (PDS) units as side-chain functionalities. Using dithiothreitol (DTT), the crosslinking of the polymer occurs through a thiol-disulfide exchange reaction among the PDS groups. The author demonstrated that the size of the nanogels could vary depending on the copolymer molecular weight, composition, and concentration. At the same time, the amount of DTT added controls the degree of crosslinking, which can also affect the release rate of the loaded doxorubicin (Dox) [36,46].

#### 2.2.6. Amine Based Crosslinking

Amine-based cross-linking is another attractive approach commonly used in the development of nano DDS including nanogel because of the high reactivity of amine groups towards several chemical moieties such as carboxylic acids, activated esters, isocyanates, iodides, and others [5]. The nanogels formed by amine-based cross-linking polymer networks have demonstrated a range of stimuli-responsive properties. For example, pH-responsive PEG-PAsp-based nanogels were fabricated via crosslinking the amine-reactive hydrophobic poly(succinimide) (PSI) with hexamethylenediamine (HMD) as the crosslinker through a nucleophilic ring-opening reaction, followed by hydrolysis of the hydrophobic core [47]. The extent of crosslinking was found to be dependent on the crosslinker feed ratio used. The responsive behavior of the nanogel was also demonstrated by the increase in nanogels size in water and the reduction in size under acidic conditions reducing the release of the protein payload [47]. pH-responsive chitosan-based nanogels is another example formed by reacting chitosan amine with dicarboxylic acid-modified chitosan in a water-in-oil (W/O) microemulsion. Nanogel showed a pH-responsive behaviour when exposed to different pH conditions, facilitating acid-triggered drug release, and this has become a very attractive approach in developing DDS for a range of biomedical applications [48]. 

## 3. Fabrication of Nanogels Using 3D Printing Technologies

The technique of creating an item from a digital file known as 3D printing can be accomplished by subtractive or additive manufacturing [49]. This technique may be used with a broad range of materials, from strong metals to elastic polymers. With the utilization of various materials and quick prototyping capabilities, the possibilities of 3D printing appear to be limitless [49]. Areas where 3D printing has been widely used include prototyping automotive parts in the aeronautical industry, bioprinting implants/organs in the medical/health care industry, developing novel drug delivery systems in the pharmaceutical industry, and fabricating clothes in the textile/fashion industry, food, and chemical industries [49]. A study reported that polymers and biomolecules can be combined to produce ad hoc nanonetworks according to the final curative aims, preserving the criteria of biocompatibility and biodegradability. Controlled polymerization, interfacial interactions, sol-gel transition, nanoscale fluid manipulation, lab-on-a-chip technology, and 3D printing are the key tactics to rely on in the future and provide novel answers to crucial healthcare issues [2]. A work focusing on the fabrication of methotrexate-loaded nanoparticles fixed in alginate-gelatine 3D printable hydrogel ink to produce a solid 3D printed tablet for oral administration was published by a researcher. The results showed that a 25 G needle was used to produce great precision (> 95%) of the 3D printed tablets, and drug release patterns were evaluated in vitro at pH 1.2 and pH 7.4 to imitate the gastrointestinal environment [50]. Research reported that 3D printing technology has been widely explored for the rapid design and fabrication of hydrogels, as required by complicated soft structures and devices. 3D printing methods are presented based on the rheology modifier of Carbomer for the direct ink writing of various functional hydrogels. It was further reported that, aside from its exceptional printing performance, mechanical properties, and biocompatibility, 3D-printed multifunctional hydrogels enable a variety of soft devices such as loadable webs, soft robots, 4D-printed leaves, and hydrogel Petri dishes. Furthermore, the Carbomer-based 3D printing technology, with its exceptional potential, offers up new opportunities for bioprinting manufacture and integrated hydrogel devices [51]. The study reported that nanogels are attractive biocompatible materials that enable the local delivery of multiple drugs. The 3D printing technology was used to precisely construct nanogel discs carrying paclitaxel and rapamycin. The 3D-printed nanogel disc rounds (12 mm diameter × 1 mm thickness) carrying paclitaxel and rapamycin evaded premature gelation during storage and the initial burst release of the drugs in the dissolution medium. In vivo 3D-printed nanogel discs permitted the successful intraperitoneal delivery of paclitaxel and rapamycin in ES-2-luc ovarian-cancer-bearing xenograft mice. According to the study, nanogels are appealing biocompatible materials that enable the local administration of numerous medications. The nanogel discs containing paclitaxel and rapamycin were carefully constructed using 3D printing technology. 3D-printed nanogel disc rounds (12 mm diameter 1 mm thickness) containing paclitaxel and rapamycin avoided premature gelation during storage as well as the medicines’ first burst release in the dissolving media. In vivo 3D-printed nanogel discs successfully delivered paclitaxel and rapamycin intraperitoneally to ES-2-luc ovarian cancer-bearing xenograft mice [52]. 

## 4. Stimuli-Responsive Drug Release Mechanisms of Nanogels

**Figure 3:**
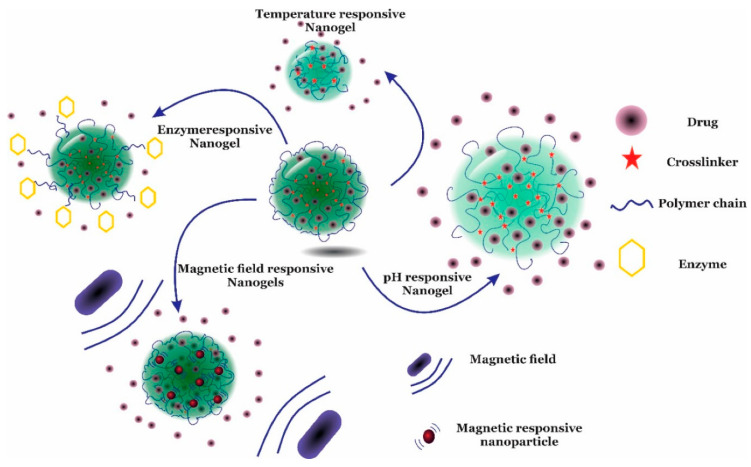
Schematic illustration of the release of drugs from a nanogel triggered by different stimuli such as temperature, enzyme, magnetic fields redox potential and pH [65].

Nanogels, like most nanocarriers, can be engineered with biomaterials that are responsive to specific microenvironmental stimuli (endogenous or exogenous) in order to facilitate intra or intercellular drug release [53]. The general idea of stimuli-responsive DDS is to optimize the localization of the adequate drug concentration to the infected cell while minimizing exposure to the healthy cells. Several stimuli conditions have been identified as the target biomarkers that can induce drug release upon exposure to a specific condition-associated infection site. The common stimuli conditions include a change in pH, enzyme concentration, temperature and redox potential within the cell, whereas some stimuli conditions are applied from the external source such as temperature, light, magnetic field, and ultrasound [10,33,53,54,55,56,57,58,59,60,61,62]. Therefore, the stimuli-responsive behavior of nanogels can be imparted by incorporating biomaterial that has responsive properties upon exposure to these stimuli conditions at the disease sites. The stimuli response of nanogels can bring about conformational or nanostructural change in the nanogel necessary for drug release at the disease site [61]. The responsive behavior is normally demonstrated in the form of rapid swelling or deswelling, or the collapse of a polymer network in the form of bond cleavage or bond dissociation. In addition, nanogel can also be easily functionalized to have a dual or multi-responsive behavior to improve their targeting ability. Based on these properties, nanogels have been recognized as one of the attractive systems over other microscopic nonresponsive gels/DDS [63]. In the following sections, attractive characteristics features of stimuli-responsive NGs will be discussed with some documented examples demonstrating their potential in achieving targeted drug delivery with minimal toxicity-related issues. A review focusing on recent advances in synthetic methodologies and the biomedical applications of “smart” NGs that can respond to single or multiple stimulus-responsive triggers was published by Preman, Namitha K. et al. (see Figure 3). This review summarizes a range of applications and the advantages of using stimuli-responsive and multifunctional NGs as the future of medicine [64].

### 4.1. Thermo-Responsive Nanogels

**Figure 4:**
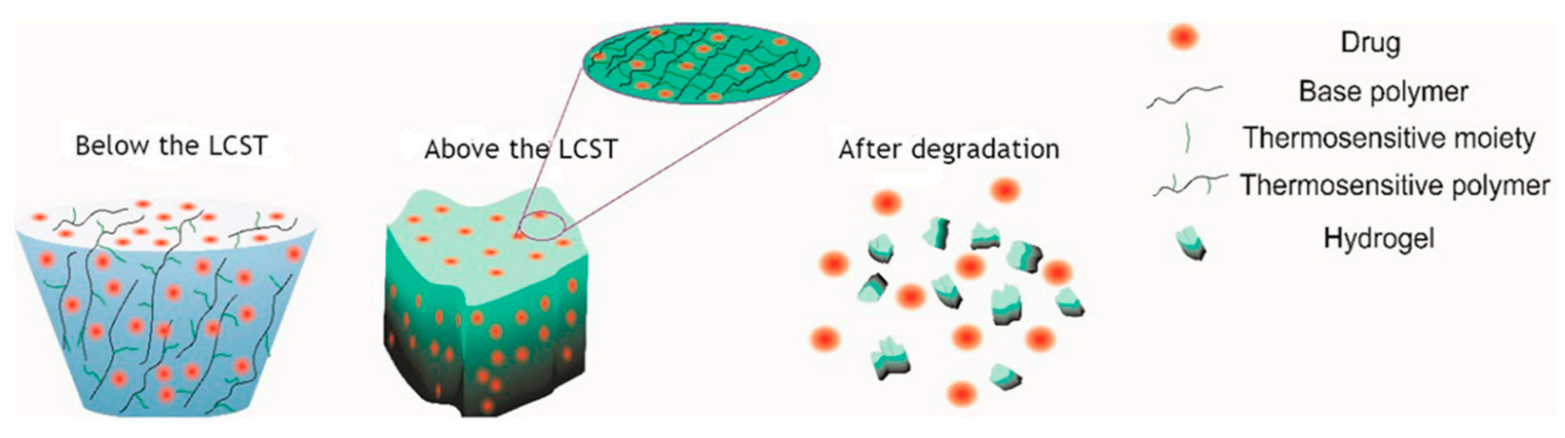
Diagram showing the degradation of the thermosensitive hydrogel below and above the LCST [65].

Some disease sites such as tumors and inflammation are characterized by elevated temperatures ranging from 40–45 °C [66,67]. These temperatures are different from the temperatures of normal cells, and nanodrug delivery systems (DDS) have been designed to recognize these temperature changes in order to facilitate targeted and controlled drug release. poly(N-isopropylacrylamide) (PNIPAM) and poly(N-vinylcaprolactam) (PVCL) are some temperature-sensitive polymers with an LCST of around 32 °C in aqueous solutions and they have been used more in designing DDS targeting disease site characterized by different temperature condition [68]. Nanogels formulated from these temperature-responsive polymers undergo swelling or deswelling at low and high temperatures, respectively, showing a volume phase transition temperature (VPTT) near-physiological temperature [68]. Temperature-sensitive nanogels can be classified into negative and positive temperature-responsive nanogels based on their volume phase transition profile in response to changes in temperature. LCST polymers such as PNIPAM are some of the most studied polymer examples used to prepare negative temperature-responsive nanogels that exhibit a hydrophilic-to-hydrophobic transition with increasing temperature [69]. Their particle size undergoes a rapid contraction when expose to higher temperature beyond their VPTT. In contrast, the upper critical solution temperature (UCST) systems undergo the opposite transition. Reported studies have shown that the LCST of PNIPAM-based nanogels can be adjusted through chain modification. The modified PNIPAM based copolymer improved the thermosensitivity properties of the nanogel showing a VPTT of 37 °C [70,71]. However, insufficient data on PNIPAM’s clinical applicability has been reported due to its in vivo non-biodegradability which can affect the toxicity profile of DDS. Galactose-functionalized poly(N-vinylcaprolactam) is one of the recent examples of an alternative polymer with thermosensitive properties that have been used to formulate thermosensitive nanogels via surfactant-free emulsion polymerization (SFEP) and reversible addition–fragmentation chain transfer (RAFT) polymerization [72]. Ghaeini-Hesaroeiye, Sobhan, et al., recently gave a summary of different thermosensitive biodegradable polymeric moieties with sharp LCST of NIPAM used in the formulation of nanogels for clinical application [65]. Figure 4 depicts an LCST-controlled degradation mechanism of a thermoresponsive nanogel to achieve targeted and controlled drug delivery.

### 4.2. pH-Responsive Nanogels

Several studies have explored the potential of pH-sensitive biomaterials in the design of DDS to achieve a site-specific and controlled drug release with respect to changes in pH levels at the disease site [62,73]. The difference in pH levels that exist between the extracellular compartments (pH = 7.4), the intracellular lysosomes (pH = 4.0–4.5) and endosomes (pH = 5.0–5.5) has been the backbone of research dedicated to developing smart DDS with pH-responsive properties for better targeting and high drug localization [74,75,76]. In addition to the benefits of using nanogel as DDS, pH-sensitive nanogels hold great potential as a therapeutic method to combat disease conditions like tumors with reduced pH levels different from physiological pH. Similarly to temperature-responsive nanogels, the pH-responsive nanogels can be shown by swelling, deswelling, or collapsing of the nanostructure depending on the type of biomaterial used during the preparation of nanogels, thereby facilitating drug release. For example, polymer materials bearing ionizable or hydrolysable bonds can bring about change (swelling/deswelling or collapsing of nanogel structure) to the nanostructure upon exposure to unfavourable pH levels at the targeted site [62]. So far, numerous pH-responsive polymers have been discovered and used to fabricate pH-sensitive nanogel for controlled and targeted drug delivery against several disease conditions characterized by abnormal pH levels [62,73,77,78,79]. For example, p(NIPAM-co-AA) was used to prepare pH-responsive nanogels to deliver doxorubicin hydrochloric acid (Dox.HCl) to treat cancer. At the simulated plasma medium of pH 7.4, nanogels showed stability with minimal drug release, whereas at lysosomal simulated medium of pH 5.0, they demonstrated an increase in drug release [80]. Another similar mechanism of release was demonstrated by Y. Li et al. using acid cleavable/degradable polymeric material. Here, Dox-loaded pH-responsive nanogel formulated from hydrophilic methoxy poly(ethylene glycol)-b-poly[N-[N-(2-aminoethyl)-2-aminoethyl]-L-glutamate] (MPEG-b-PNLG) and hydrophobic terephthalaldehyde (TPA) as a cross-linker was reported. This polypeptide-based pH-responsive nanogel shows stability at 7.4. In contrast, at tumoral simulated acidic conditions (pH~6.4), the nanogel degraded, causing a rapid release of DOX due to the breaking of the acid-cleavable imine bond within the polymeric network system [78]. The above and other recent studies reported in the literature suggest that pH-responsive nanogels could be one of the most lucrative strategies to address the limitation associated with treating disease conditions characterized by abnormal pH levels [79,81,82,83,84].

### 4.3. Photo-Responsive Nanogels

Designing nanogel based on photoresponsive polymers is one of the promising but understudied research topics. Biomaterial bearing chromophore groups that can absorb light, especially within a window of 650–900 nm, in the near-infrared(NIR) range, have been used to develop a nanosystem of this nature due to minimum absorbance of NIR light by tissue and skin [54]. NIR irradiations are regarded as weak energies to induce direct isomerization, bond cleavage, or caging/uncaging compared to using high-energy irradiation like ultraviolet-visible (UV) or visible light [85]; however, UV or visible light’s direct use is associated with tissue damage and cannot penetrate beyond the skin into deep tissues [37]. Therefore, NIR light remains the only option in developing light-stimulated hybrid nanogels with good penetration through the skin and other tissues to about a centimetre in depth. Photoresponsive nanogels can be divided into two categories depending on the biomaterial used during their formulation [59,86]. The first is a system that can undergo phase transition that induces structural or polarity change of polymer functional groups upon exposure to light of the appropriate wavelength. The second type is a hybrid system composed of noble metal NPs (Au and Ag) and a temperature-sensitive polymer network [87]. In this case, metal elements within the nanogel system can absorb light and create localized heating, which can then induce phase transition in the temperature-sensitive polymers, facilitating drug release [87]. The use of these metals, particularly Au-NP for drug delivery systems, has been attributed to their low toxicity-related issues, lack of self-quenching, and large optical cross-sections. In addition, they exhibit high stability at the nanoscale and possess the unique surface chemistry necessary for surface modification using organic molecules, polymers or biomacromolecules via the common Au–thiol bond [88,89]. Photothermal therapies have been produced using light-responsive Au-NP-containing nanogels. The photoactive nanogels can be triggered to release the drug once reaching the disease site using externally delivered photoirradiation. For cancer treatment, PEGylated nanogels containing Au-NP within the core of cross-linked networks of poly[2-(N,N-diethylamino)- ethyl methacrylate] are one the examples of photo-sensitive nanogels reported [90,91]. These nanogels showed high toxicity against cancer cells upon irradiation with Ar^+^ laser promoting intracellular heat generation by NGs, which resulted in selective and non-invasive cancer photothermal therapy. This indicated that light-sensitive nanogels with metal NPs could be used for both drug delivery and release and localized heating for thermal therapy [92]. Hence the use of hybrid nanogels in a dual-mode therapeutic strategy has been found to improve therapeutic efficacy.

### 4.4. Magnetic-Responsive Nanogels

Nanogels containing magnetic nanoparticles (NPs), such as Fe_2_O_3_ or Fe_3_O_4_ NPs, represent another type of hybrid system that can be triggered by applying external stimuli, such as magnetic fields [55,93]. Superparamagnetic NPs are incorporated in drug delivery systems with magnetic fields that can penetrate the body tissue non-invasively. Magnetic field-sensitive NGs undergo a similar responsive mechanism as in photo-responsive nanogels to potentiate high drug localization at the site of the infection when the external magnetic field is applied. This approach was reported by *Adriane* et al. who monitored an auricular VX2 tumor over two weeks after injecting a rabbit with magnetic poly(vinyl pyrrolidone) nanogels loaded with the chemotherapeutic Bleomycin A5 Hydrochloride (BLM) as the model drug with a permanent magnet placed directly over the surface of the tumor for 24 h. After two weeks of treatment, the tumor showed a significant size reduction, attributed to combined treatment with nanogel particles containing BLM and the permanent magnetic field [94]. The magnetic poly (NIPA) DOX-loaded NG is also another example reported by Purushotham et al. Upon application of AMF on magnetic poly(NIPA) NG, the heat produced increased the temperature to higher than its volume phase transition temperature (VPTT), causing a sudden disintegration of NG, facilitating rapid doxorubicin release [95]. Similarly to photo-responsive nanogels, nanogels containing magnetic NPs can also be used for thermal therapy since they can generate heat upon exposure to an alternating magnetic field (AMF) [96,97]. These benefits highlight the superiority of using these approaches when designing functionalized nanogels for a better therapeutic outcome as compared to non-responsive nanogels.

### 4.5. Ultrasound-Responsive Nanogels

Ultrasound-sensitive polymeric systems have been introduced as an alternative in achieving spatiotemporally controlled drug release at the desired location with minimal harmful systemic effects [56,98]. Ultrasound, including high-intensity, focused ultrasound, has been widely used for biomedical applications, especially in tumor therapy. The use of ultrasound can be attributed to its non-invasive nature, ease of accessibility, lack of ionizing radiation residues, cost-effectiveness, controllable spatiotemporal effect, and high patient acceptability [99]. As ultrasound-responsive polymeric systems, many new nanodroplets, nanobubbles, nanomicelles, and nanogels have been produced. To enhance the thrombolysis effect, a urokinase-type plasminogen activator (uPA) was loaded into hollow nanogels prepared by a one-step ultrasonic spray reaction of glycol chitosan and aldehyde capped poly(ethylene glycol) (OHC-PEG-CHO). The in vivo study demonstrated that uPA-loaded nanogels had a prolonged circulation time as compared to the bare urokinase and increased the protein release rate under ultrasonic conditions of 2 MHz. In another study, the ultrasonic responsiveness of nanogels was demonstrated using adriamycin (ADM)-loaded gelatin-based nanogel functionalized with fluoride anion (ADM-GNMF). At a frequency of 20 kHz, the change in size and ultrasound-triggered drug release were observed [56].

### 4.6. Multi-Stimuli-Responsive Nanogels

Although the co-delivery of multiple drugs using one delivery system has demonstrated superior therapeutic benefits when compared to the delivery of a single drug, controlling the release of each drug from a single multi-delivery system remains a challenge [100,101]. Researchers within the field of nanomedicine have already attempted to overcome this limitation by introducing layer-by-layer assemblies in which programmable and sequential release can be achieved [102]. Particularly, multi-sensitive nanogels designed to respond to more than one stimulus formulated using polymer materials with different responsive properties via several preparative methods have been reported [103,104,105,106]. They can be designed to simultaneously respond to all stimuli. However, mutual interference between different stimulus-responsive biomaterials has been one of the drawbacks of such delivery systems. This shortcoming was addressed by adopting a sequential polymerization method to prepare multi-responsive nanogels; in this way, stimulus-responsive polymer components are independent of one another [62,107]. pH- and temperature-responsive nanogels are commonly studied individually and as a combination of stimuli-responsive systems. There are several disease conditions in which changes in pH and temperature change occur simultaneously. A DOX-loaded hollow nanogel with dual sensitivity to changes in pH and temperature for efficient intracellular drug delivery was reported by Chiang et al. The system was prepared using the combination of copolymers such as acrylic acid (AAc) and 2-methacryloylethyl acrylate (MEA) units with other chains, either poly(N-isopropylacrylamide) P(NIPAm) or grafted monomethoxypoly(ethylene glycol) (mPEG). The AAc/DOX complexes showed stability at neutral, whereas under acidic conditions (pH 5 at 37 °C), there was reduced ionization and size of the nanogels. The cytotoxicity effect of the DOX-loaded hollow nanogels against cancer was greater compared to free drug [108]. Table 1 below highlights some examples of multi-stimuli-responsive nanogels and their biomedical application.

## 5. Passive Targeting of Nanogel

**Figure 5:**
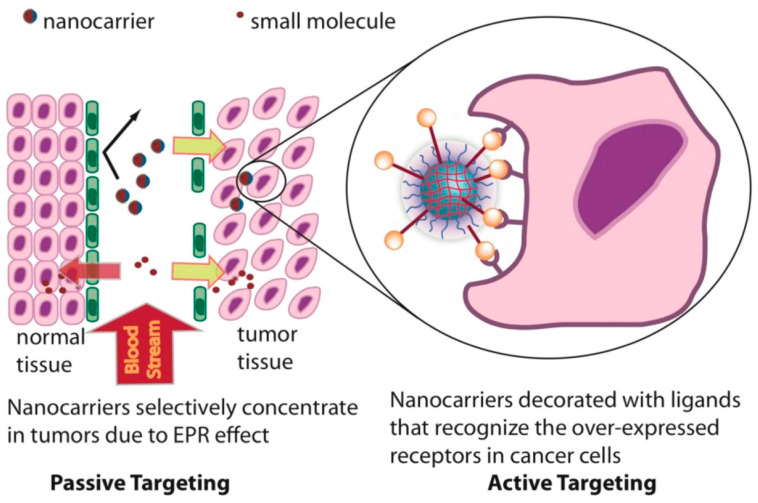
The EPR effect representing passive targeting mechanism and ligand functionalization representing active targeting mechanism [119].

Passive targeting using delivery vehicles primarily relies on the enhanced permeation retention effect (EPR). Matsumura and Maeda were the first to demonstrate this concept in 1986 after observing the hyperpermeability of vasculature around cancer cells, with wide openings in between the cells, which permits nanoparticles to build up in the tumor (see Figure 5) [116]. As a result of the EPR phenomenon in cancer, it is possible to target diseased cells simply by adjusting the delivery system’s size. Therefore, supramolecular systems with controllable size produce more therapeutic effects against disease conditions like cancer and arthritis [116,117]. In addition, the size of a delivery vehicle, the method of administration, and the pathway it uses to reach the target site influence the rate of cellular internalization. Gene therapy has played a major role in improving the quality of life of cancer patients. Recently, non-virus polymers with low immunogenicity, tailorable structure, and tuneable size have become an alternative to promote gene transfection. PNIPAM/PEI nanogels with thermosensitive properties were developed from PNIPAM as the core and cationic PEI as the outer shell, demonstrating the non-toxicity of PEI with enhanced polyplex stability and cellular targeting. Laser light scattering was used to evaluate the polyplex properties of the nanogel, including size and surface potential, thermo-sensitivity, and serum stability. The nanogel’s in vitro transfection efficiency was roughly two folds higher when compared to that of PEI, and another two folds increased due to passive cell targeting as T > VPTT. Inverse fluorescence microscopy, confocal laser scanning microscopy, and a flow cytometer were used to demonstrate the nanogel’s improved in vitro cellular uptake, gene transfer efficiency, and their corresponding mechanism. The biodistribution and considerable intratumor accumulation within the Balb/c nude mice xenograft tumor type were also studied. The overall result from this study proposed that nanogel can significantly suppress tumor growth compared to the “gold-standard” transfection agent poly(ethylenimine) (PEI), which indicated its high potential as a gene therapy [118].

## 6. Active Targeting and Corresponding Modification of Nanogels

While passive targeting remains the standard mechanism by which most DDS deliver the drug to the targeted site, active targeting has been the alternative strategy to improve the targeting ability of some nonresponsive DDS that depend solely on the EPR effect [37]. Like most of the DDS, nanogels can be functionalized small molecules/ligands capable of recognizing and having high binding affinity to specific receptors primarily overexpressed by the infected cells (see Figure 5) [120]. Antibodies, fragments, and recombinant polypeptides, including single-chain antigen-binding fragments (scFv), nutrients, receptor ligands, hormones, mediators, peptides, nucleic acids, and aptamers, polysaccharides, and small molecules, form part of ligands that have been or may be used for this purpose [10,121,122]. Active targeting has been regarded as or limited to targeting cell-surface receptors specific to the targeted region [123]. However, as highlighted in Section 4 with some examples, a variety of biomarkers, such as extracellular enzymes (e.g., matrix metalloproteases) and microenvironmental parameters (e.g., pH, reactive oxygen species, temperature, etc.), have been used to promote targeting and control drug release (see Section 4) [124,125].

### 6.1. Small-Molecule Conjugation

The ligand binding targeting approach has been used quite extensively to treat cancer-infected tissues while minimizing exposure of the cytotoxic drug to healthy cells [126]. Folic acid surface functionalized DDS is one example reported with some clinical applications showing an improved therapeutic response against cancer-infected cells. For example, folate receptors (FRs) are overexpressed in cancer cells such as ovarian carcinomas and other tumors that are common to humans, and their expressions in healthy tissues are very low [127,128]. Therefore, the variation in the expressions of FRs becomes a maker or target molecule to afford the treatment of such malignance disease [129]. Furthermore, the use of nanosystem surface-functionalized with folic acid can improve the internalization of the drug by the tumor via FR binding [130]. Folate-decorated nanogel is one example of a cell receptor targeting method with a small-molecule conjugation to enhance its therapeutic effect against ovarian cancer. The optimized FA-nanogels were shown to enhance the accumulation of the encapsulated drug in the cell population with high expression of FR. This was demonstrated with the enhanced anti-tumor therapeutic outcome of CDDP anti-cancer drug, delivered to a xenograft tumor using these FR-targeted nanogels and reduced renal toxicity. Overall, this study demonstrated the capacity of nanogel for the selective delivery of anti-cancer drugs to improve their therapeutic efficacy [130].

### 6.2. Peptide Conjugation

Peptides are well-known for their low immunogenicity and biological functions that closely resemble or outperform natural proteins [131,132]. As a result, designing nanomedicines with active cell-targeting peptides is one approach that can improve cell penetration ability and stimulate selective cellular uptake via receptor-mediated endocytosis [123,133,134]. Nanogels decorated with peptides are among the nanosystems reported to improve the targeting and therapeutic efficiency of drugs against various diseases such as cancer. A peptide-modified nanogel for siRNA targeted delivery for gene silencing was reported by Blackburn et al. [135]. In this study, hepatocellular (Eph) A2 receptor was identified as the marker or targeted molecule to improve tumor-targeted delivery. Thereafter, they designed peptides of 12 amino acids (YSAYPDSVPMMS or YSA) which can mimic ligand ephrin-A1, which has a binding affinity to the erythropoietin producing (Eph) A2 receptor. In addition to high loading capacity for siRNA, peptide functionalized core/shell pNIPMAm nanogel improved site-specific delivery via ligand-receptor binding mediated endocytosis to the cytoplasm of ovarian cancer cells. Furthermore, their ability to silence genes, peptide-functionalized nanogels were complimented by minimal levels of toxicity together with high therapeutic efficiency in the presence of the serum-containing medium. The result also suggests a need for a better understanding of the basic mechanisms of nanogel endosomal release. Lastly, the author expressed the need to further evaluate these outcomes using in vivo animal models for delivery and silencing in order to understand its potential in clinical application [135]. A surface-conjugated thermo/pH-responsive nanogel with anti-collagen IV peptide used to deliver rapamycin (RAPA) was also reported as a promising approach for restenosis therapy. Neointimal hyperplasia is reduced more effectively with peptide-coupled nanogels than with non-targeting nanogels. In addition to its targeting ability, its stimuli-responsive properties showed a desirable intracellular release profile of RAPA with a significant reduction in the adverse effect of RAPA. The increased restenosis preventative efficacy was also attributed to the selective release of RAPA on the injured artery sites, demonstrating the nanosystem’s potential to deliver a systemically tailored treatment [136].

### 6.3. Antibody Conjugation

Apart from using small molecules or peptides, surface conjugating with antibodies for targeting purposes is also another alternative strategy used to improve the interaction with particular cancer cells while limiting side effects on healthy tissue [137,138]. Surface-modified nanogels with antibodies to achieve higher targeting with precision have shown some positive results in treating cancer-infected cells [139]. This technique makes use of membrane proteins overexpressed in tumor cells to create antibodies that can later be used as nanoparticle vectors [140]. These vectors have a high binding affinity to their targets, forming complexes that are internalized into the cell through receptor-mediated endocytosis. The drug loaded into the nanoparticle can diffuse directly through the cellular membrane, increasing the drug’s cellular penetration and thus enhancing its therapeutic efficiency [141]. Anti-CD4 conjugated mertansine-loaded nanogels, for example, were designed to target lymphocytes (CD4+ T cells) cells with high expression CD4 glycoprotein on their surface. According to this report, the antibody conjugation enhanced the cellular uptake of nanogel (NG) by CD4+ T cells while decreasing their nonspecific uptake by CD4 lymphocytes. Furthermore, at 17 ng/mL antibody concentration, the mertansine-loaded conjugate inhibited cell growth in T-lymphoma cell lines in a dose-dependent manner. In contrast, the same level of cell growth inhibition was observed when using antibody–drug conjugate (ADC)-type anti-CD4 formulations but at a slightly higher concentration of 1.8 µg/ mL. These findings indicated that NG antibody conjugation could efficiently transport a large payload to a specific target with a much lower antibody quantity required to achieve a similar result [142].

### 6.4. Bio-Membrane Camouflaged

Cell membrane coating technology is a method for reproducing cell membrane features in a biomimetic manner, and it is currently a hot topic in nanoscale biomedicine. Cell membrane surface-decorated nanoparticles (NPs) are one of the ways cell membrane properties are merged with NPs’ properties to achieve superior therapeutic outcomes [143]. Coated NPs achieve effective and long circulation in vivo, enabling the execution of specified tasks. Although cell membrane-camouflaged NPs have obvious benefits, considerable work is still to be done before being used in clinical practice [144]. The use of cells in drug delivery has contributed towards advancements made in cancer therapy. For example, bioinspired nanogels with stem cell membrane camouflage have been produced for targeted photodynamic therapy of lung cancer. Nanogels were coated with stem cell membrane vesicles as the outer shells with the inner core consisting of hydrophobic photosensitizer, chlorin e6 (Ce6)-loaded gelatin nanogels (Ng) (Ng/Ce6) denoted as nanogels (Ng/Ce6@SCV). Using (SCV) Ng/Ce6@SCV promotes Ce6 internalization by cells and the generation of ROS within the tumor cells upon irradiation by the NIR laser, inhibiting the proliferation of A549 tumor cells in vitro. Stem cell membrane camouflage Ng/Ce6@SCV demonstrated significant targeting ability with high accumulation and prolonged retention within tumor tissues by avoiding immune response activation. An in vivo anti-tumor study demonstrated that Ng/Ce6@SCV had a superior anti-tumor effect after NIR irradiation, effectively inhibiting primary tumor growth with minimum adverse effects. [145]. In summary, the findings indicate that the polyphosphoester-based bioinspired DDS has potential as one of the alternatives and effective PDT methods for cancer treatment.

## 7. Application of Nanogels for the Delivery of Low and High-Molecular-Weight Chemotherapeutic Agents

Nanogels attracted a lot of attention as nanoscopic drug carriers, especially for delivering bioactive mediators to specific sites or at certain times. The versatility of polymer systems and the ease with which their physicochemical properties can be changed have resulted in versatile nanogel formulations. Nanogels offer exceptional stability, drug loading capacity, biological consistency, strong penetration ability, and the ability to respond to environmental stimuli. Nanogels have shown great potential in the field of nanomedicine as a superior DDS, particularly in gene and chemotherapeutic drug delivery, diagnostics, organ targeting, and in many more applications. Table 2 summarises the biomedical applications of nanogel in chemotherapy.

### 7.1. Small-Molecule Delivery

Designing an effective drug delivery vehicle for many malignancies such as cancer is still met with many challenges related to the body’s system complexity. However, significant progress has been made in developing “smart” DDS with unique properties, particularly nanogels for the delivery of small therapeutic agents for a range of disease conditions [9,152]. Several reports have demonstrated the use of nanogels to deliver small molecules. The work of Du et al. is one example; they created a pH-responsive charge-switching nanogel to enhance tumor cellular uptake and intracellular doxorubicin (Dox) release. The negatively charged nanogels were made by first preparing poly (2-aminoethyl methacrylate hydrochloride) (PAMA) and then treating it with 2,3-dimethylmaleic anhydride (DMMA). In this example, the release rate of Dox from nanogels increased as the pH value decreased. The results from this study also revealed that the cell viability of MDA-MB-435s cells treated with Dox-loaded nanogels was low at pH 6.8 compared to pH 7.4 and when using free Dox at both pH conditions [75].

Another study published in 2017 by Luan et al. demonstrates the pH-responsiveness of hyaluronic-acid-based nanogels for tumor-targeted Dox delivery. A similar trend from the above study was also observed with an accelerated Dox release under acidic simulated tumor conditions. The Dox-loaded nanogels displayed much higher in vitro cytotoxicity than free Dox. Confocal microscopy indicated that tumor cells cultured with pH-sensitive Dox-loaded nanogels from 3 to 12 h had a substantial increase in Dox in their nucleus. The increased anticancer effectiveness of Dox-loaded pH-sensitive nanogels was confirmed using in vivo treatment trials on mice, in which tumor volume evolution was recorded and tumor tissue cell death and proliferation were studied [146]. Recently, glutathione-sensitive double cross-linked PEGylated PMAA-based nanogel (PMAABACy/Fe(III)-co-PEGMA) was developed to improve Dox intracellular delivery. This strategy uses GSH levels within and outside tumor cells to identify the target and effectively release Dox within the tumor cell. A fluorescence microscope was used to demonstrate the cellular internalization of nanogels and the intracellular Dox release by nanogels. At the same time, flow cytometric measurements at various time intervals of incubation validated the intracellular release of Dox. The red fluorescence signified Dox presence, and with an increase in incubation time, the intensity of red fluorescence increased indicating Dox-loaded nanogel cellular uptake. This information was further verified with results from flow cytometric assays. Compared to free Dox, Dox-loaded PMAABACy/Fe(III)-co-PEGMA_950-2_ demonstrated a slow intracellular release within 8 h which further translated to excellent anticancer activity against tumor cells [147].

### 7.2. Bio-Macromolecule Delivery

Several biotherapeutics, such as proteins and DNA, are employed as medicines and have their target inside the cell. However, challenges facing their delivery to specific areas within cells have been associated with enormous molecular size, difficulty passing through cellular membranes, and susceptibility to enzymatic and chemical destruction. Nanogels, because of their flexible three-dimensional polymer network structure, became a suitable carrier for this type of delivery, since they can accommodate all sizes of biotherapeutics, proving a safe passage through the cell membrane and a shield from degradation and allowing for cell internalization.

#### 7.2.1. Proteins Delivery

Several disease conditions, including cancer, autoimmune illnesses, and metabolic disorders, are also being treated with therapeutic proteins. Reduced stability and short circulation half-lives following parenteral injection are two major issues that restrict the efficacy of protein therapies [153]. High doses are given frequently to maintain effective protein concentration to improve their therapeutic efficacy. However, such uncontrolled and large dose administration leads to nonspecific protein delivery throughout the body, resulting in unwanted side effects and a lower quality of life for patients [153]. As a result, efficient protein delivery systems are urgently needed to improve bioavailability and allow for a tailored controlled release profile. Self-assembled protein-loaded pH-sensitive cholesteryl pullulan-based nanogels were developed to retain the encapsulated protein at the physiological pH of 7.4 and release protein under reduced pH conditions. These discoveries suggested that stimuli-sensitive, self-assembled nanogels can promote a sustained release of protein based on the rate of degradation of the cholesterol-pullulan grafting moiety [154].

#### 7.2.2. Nucleic Acid Delivery

Gene treatment introduces foreign genomic material into specific host cells to acquire a therapeutic benefit by repairing existing dysfunctions or sustaining individual cells with new functions [155]. Due to their adverse pharmacological features, such as high molecular weight, hydrophilicity, negative charge, and difficulty permeating the negatively charged and lipophilic-layered cell membrane, nucleic acids are difficult to give to their intracellular targets. Furthermore, in vivo, nucleases in the blood rapidly destroy unprotected nucleic acids [156]. Therefore, polymeric nanogels present a promising delivery platform for DNA or RNA molecule therapies. Furthermore, owing to its physicochemical properties, decorated siRNA can reach specific tissues. A nanogel-based delivery device was used to carry out the siRNA therapy. This strategy paves the way for future functional oligonucleotide therapeutics. The tetrahedral DNA-based (TET) nanogel was used as a nonviral vector for siRNA assembly and delivery protection. This method may effectively protect the ribonuclease from degradation and cell transfection in vitro and in vivo, indicating that it could be a promising platform for combining numerous devices for enhanced efficiency [157].

Even though the available data on cancer therapy highlights the potential of nanogels as a versatile drug delivery system, it is still presented with some limitations, which suggests that currently, there is a lack of efficient treatment available for cancer. Indeed, it is impossible to achieve a desirable outcome with a single therapeutic approach because of this disease’s biological complexity. Even though several studies have reported delivery systems delivering a specific drug or gene/macromolecules to improve their efficacy, they are still faced with some restrictions that delay their clinic application. A considerable number of studies have indicated the need to develop an alternative system to improve the inadequate suppression of any disease condition by single-drug delivery systems. Compared to a single-drug method, a co-delivery system has proven to be a promising strategy for enhancing the target response or achieving a synergistic/combined impact.

## 8. Nanogel in Combinational Chemotherapy

Cancer is still considered one of the leading causes of death across the globe with limited treatment options. Combination therapy has been adopted as one of the alternative treatment approaches to fight cancerous disease with some successful clinical applications in patients with breast and ovarian cancers [158]. The success of this therapeutic approach can be attributed to its capacity to produce synergistic anticancer effects, decrease individual toxicity related to a specific drug, and lower multi-drug resistance development. However, because different therapeutic agents have distinct physicochemical properties, achieving the optimum utility of an individual drug at concentrations below their toxic levels remains a challenge [159]. Nanotechnology therapies based on co-delivery have been identified as a viable technique, bypassing several biological, biophysical, and biomedical barriers that the body erects to prevent anticancer medications from reaching their target tissues [160]. Combining multiple bioactive agents within a single nanocarrier can reduce the frequency of administration, minimize drug-related adverse effects, and improve patient compliance. Nanogel is one of the nanoscale structures, as discussed in previous sections, that can easily be manipulated or transformed with features suited for combinatorial encapsulation of pharmaceuticals with varying pharmacokinetic properties. Several researchers have investigated the combination index of Dox with other chemotherapeutic agents using different types of nanogels systems. In 2017, Wu, Haiqiu, et al. reported the design of stimuli-responsive polymeric nanogels based on poly(acrylic acid) for the codelivery system of Dox and cisplatin to reduce drug use resistance-related issues. The synergistic effect of CDDP/Dox combination chemotherapy was achieved by encapsulating both drugs into the nanogels, demonstrating superior in vitro antitumor activity, and making it an effective strategy to treat drug-resistant tumors. Self-targeting hyaluronate (HA) nanogels (CDDPHANG/Dox) were developed in another study as a method to reverse drug resistance in MCF-7/ADR breast cancer cells by synchronizing the pharmacokinetics, intertumoral distribution, and intracellular release of topoisomerase II inhibitor doxorubicin (Dox) and DNA cross-linking agent cisplatin (CDDP). The HA nanogel showed CD44-positive tumor targeting, MDR-reversing, pH-responsive release, fluorescence/micro-CT dual imaging, and synergistic chemotherapeutic actions, indicating that it has a lot of potential in future clinical drug-resistant breast cancer therapy [148]. Nanogels are also appropriate nanoplatforms for combination therapy, according to these findings.

### 8.1. Photo Induced Chemotherapy

Phototherapy has been proven to be an effective therapy for treating primary tumors since the 20th century. It differs from conventional chemotherapy, radiotherapy, and gene therapy because of its non-invasiveness, ease of operation, low drug resistance, high temporal–spatial resolution, and low adverse side effects [161,162,163]. The process of cancer phototherapy mainly relies on delivering the light-absorbing therapeutic agent to the tumor site to achieve a precise spatiotemporal control of drug release upon exposure to specific light. Phototherapies can be categorised into photodynamic therapy (PDT) and photothermal therapy (PTT), and they are characterized by the different mechanisms through which they induce cancer cell death. In photothermal therapy (PTT), light is converted into hyperthermia for the direct ablation of cancer cells, causing extremely low damage to surrounding healthy tissues. On the other hand, PDT employs photosensitizers and light to produce cytotoxic reactive oxygen species (ROS), such as singlet oxygen (O_2_), which causes oxidative stress in cancer cells, inducing apoptosis [164]. Despite the positives of phototherapy against cancer, laser intensity decreases with the depth of the biological tissue, reducing the ability to destroy the tumor. The heat created in the tissue is distributed unevenly, causing damage to other tissues. Based on these limitations, many nanomaterials for drug delivery are extensively explored as agents for cancer phototherapy [164,165]. Due to the nature of chemotherapy, a DDS such as nanogels that combines phototherapy and chemotherapy can effectively overcome the concerns mentioned above. In the next section, further details on the applications of photothermal chemotherapy and photodynamic chemotherapy are discussed.

#### 8.1.1. Photothermal Chemotherapy

Photothermal therapy alone has been associated with uneven hyperthermia distribution, leading to incomplete tumor ablation, whereas chemotherapy can develop serious side effects and drug resistance [166]. Therefore, merging photothermal therapy and chemotherapy (photothermal-chemotherapy, PT-CT) has recently emerged as a feasible alternative for cancer treatment with improved therapeutic efficiency and reduced side effects [149,166,167]. PTT involves using photothermal conversion agents (PTAs) to generate enough heat upon exposure to near-infrared (NIR, 700–2500 nm) light to increase the temperature of the surrounding environment and trigger tumor inhibition and ablation [149,168,169]. The use of PTT over other therapies can be attributed to its advantages, including minimal invasiveness and high selectivity in combination with minimal side effects, since the laser irradiation parameters used, such as location, wavelength, irradiation time and intensity of light, can be controlled [169].

A combined chemo-photothermal therapy based on nanomaterials has shown superior therapeutic effect over single cancer treatment. Since nanomedicine can selectively accumulate into the tumor site, the co-delivery of cytotoxic drugs and hyperthermia can produce a synergistic effect to improve cancer treatments [170]. This combination therapy can achieve a synergistic therapeutic index via several mechanisms: (i) promoting high cell membrane permeability; (ii) enhancing drug cytotoxicity; (iii) triggering drug release at the targeted region. However, the limitation facing the nanoformulation applications developed so far is the lack of active drug delivery properties, which leads to insufficient drug concentration in tumor tissue. Nanogels have demonstrated the ability to overcome problems associated with other delivery systems because of their previously mentioned unique physicochemical properties. Recent studies have demonstrated the use of pNIPAAm-based nanogels with different photothermal conversions for both photothermal therapy and thermally controlled drug release. In 2018, Chang, Ray, and Wei-Bor Tsai reported thermo-responsive pNIPAAm-based nanogels co-polymerized with N-(hydroxymethyl)acrylamide (NHMAAm) for regulating LCST in order to adjust the responsive temperature. Photosensitive sodium copper chlorophyllin (SCC) was incorporated into nanogels which, upon exposure to the 532 nm green laser, showed the generation of significant heat to cause cell death. To demonstrate photothermal-chemotherapy, nanogels were loaded with Fluorouracil (5-FU) which, when exposed to the green laser, released the drug at high concentration to enhance cell death to a maximal extent [150]. Another study reported by Zhang, Weili, et al. also reported crosslinking of NIPAM monomer with N,N′-bis(acryloyl)cystamine reducible linker and in situ polymerized with graphene oxide (GO) to produce biodegradable hybridized nanogels (PG) with high biocompatibility [149]. The combination of nanogel redox sensitivity and GO-induced heat conversion for accelerated drug release at the tumor site. Both studies indicated that nanogels could achieve chemo-photothermal synergistic therapy, especially in treating cancers to reduce the development of multidrug resistance (MDR).

#### 8.1.2. Photodynamic Chemotherapy

Photodynamic therapy (PDT) is considered a minimally invasive treatment and is currently employed as an alternate treatment for cancer control in clinics [171,172]. PDT induces cell death by producing oxidant species (radicals, singlet oxygen, and triplet species) generated by photosensitizer (PS) molecules when activated by light of a specific wavelength [172]. However, challenges such as poor penetration of light (particularly UV and visible light) into biologic tissue limit the use of this therapeutic method only to the treatment of tumors on or just beneath the skin and the exterior linings of internal organs and cavities [173]. In addition, nonspecific distribution of PS in the body may lead to inevitable side effects. To overcome these limitations, using multifunctional DDS for combination therapy using PDT and chemotherapeutic agents has shown great potential to scavenge tumor cells and reduce MDR effectively. Nanogels have demonstrated the ability to accommodate chemo-photodynamic combinational therapy as a smart multifunctional delivery platform. The photosensitizer (tetraphenylporphyrin zinc [Zn-Por]) was combined with histidine to create a pH-responsive metallo-supramolecular nanogel (SNG) that can recognize an acidic tumor location and release both an anticancer treatment and a photosensitizer to kill the lesion cells. SNG contains Zn-Por moieties that retain photosensitivity in the visible wavelength range and can generate active oxygen species for photodynamic treatment [174]. Therefore, the drug-loaded SNG serves as both a chemotherapeutic and photodynamic treatment platform.

### 8.2. Combinatorial Chemo-Immunotherapy

**Figure 6:**
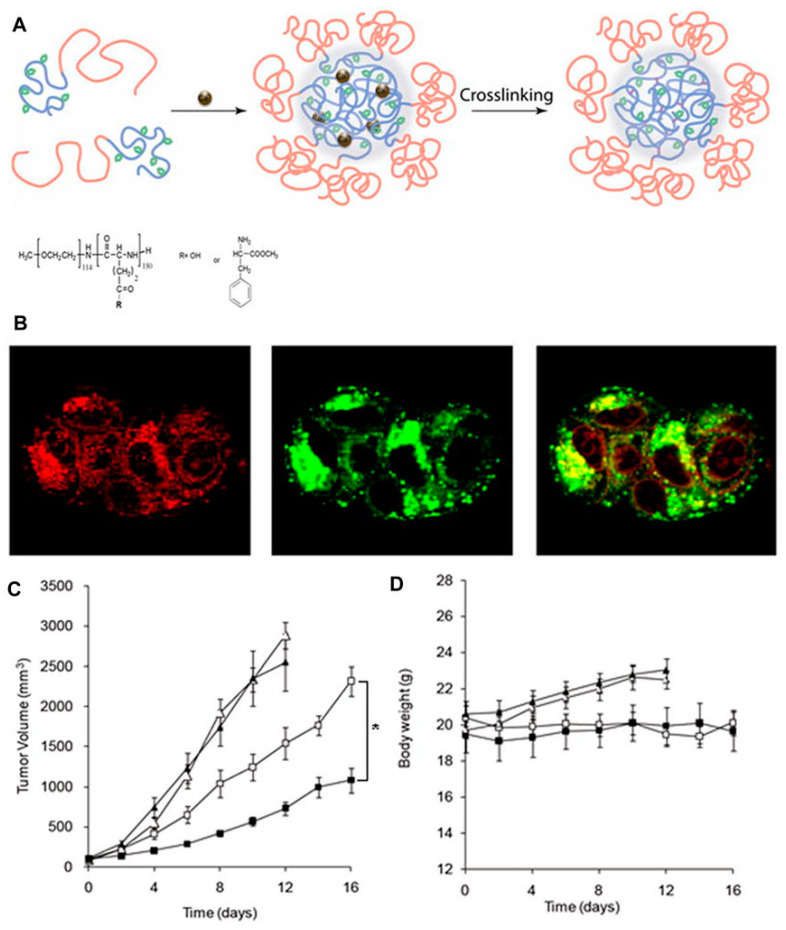
Synthesis and biological characterization of nanogels. (**A**) Schematic diagram of nanoparticle self-assembly. (**B**) Localization of DOX-loaded nanogels in cells. Effects of DOX-loaded nanogels on the tumor volume (**C**) and body weight (**D**) [178].

Chemotherapeutic failure to eliminate cancer may be attributed to fast mutations within tumor cell subgroups that avoid cytotoxic medicines. The presence of lymphocytes within the tumor microenvironment causes the suppression of anti-cancer immune responses, allowing them to grow, develop, and metastasize more easily [175,176]. However, protein therapies, such as cytokines and antibodies, can stimulate the immune system to produce an effective anticancer response. Therefore, combination chemo-immunotherapy methods can synergize therapeutic effects against cancer cells and inhibit drug resistance [177]. Although it has demonstrated feasibility for clinical application, it still faces limitations, such as short drug and protein half-lives, systemic toxicity, and variable in vivo pharmacokinetics and distribution, which reduce anti-cancer efficacy [176,177]. The application of nanomedicine to deliver chemotherapies combined with immunotherapy is recognized as a promising, non-toxic, and efficacious tumor treatment strategy [60]. Folate pH-degradable PVA nanogels were successfully developed for the simultaneous administration of docetaxel (DTX) and N9 to boost cancer chemo-immunotherapy. The drug-loaded nanogels not only killed tumor cells directly with DTX but also triggered immunogenic cell death (ICD), allowing cytotoxic T lymphocytes to accumulate in the tumor. Combining with N9 boosted CD8+ T-cell and NK-cell intratumoral infiltration while inhibiting MDSC infiltration, lowering IDO1-mediated immunosuppression [151]. Recently, another study reported the use of reduction-responsive polypeptide nanogel for synchronous delivery of an ICD inducer DOX and an immune-regulating agent 1MT into the tumor site to enhance synergistic antitumor efficacy (see Figure 6). Using flow cytometry, the bare DOX+1MT and NG/(DOX+1MT) immunomodulatory effect in 4T1 mouse tumors, TDLNs, and tumor-infiltrating lymphocytes (TILs) were investigated. Using both DOX+1MT and NG/(DOX+1MT) therapies showed enhanced antitumour activity of CD8+ T cells through reduced recruitment of Treg and MDSC [175]. In addition, an in vivo study using NG/(DOX+1MT) demonstrated superior immune modulation compared to bare DOX+1MT, which can be attributed to effective drug transportation to the tumor site and high suppression of IDO expression. These findings showed that chemoimmunotherapy significantly inhibited tumor development while having no obvious side effects, indicating that it has a lot of potential in clinical cancer therapy.

## 9. Nanogel Toxicity and Nanotoxicology

Nanotechnology’s application in medicine, particularly for drug delivery, is growing rapidly, and more biomaterials are being introduced, especially for cancer therapy [179,180]. However, the use of drug delivery systems is to lessen drug toxicity and increase biocompatibility [180]. Recent research studies have reported some potential risks in using these carriers. Hence a nanotoxicology study was adopted to assess the effects of nanodevices and nanostructures in living organisms [181]. Even though nanotoxicology and nanosafety have been topics of interest for nearly two decades with many research articles published on the subject, there is still a knowledge gap, and approaches to standardize nano risk assessment [182]. These include sub-optimal in vitro models, a lack of in vitro–in vivo correlations, deficiencies in both material purity and physicochemical characterisation, and heterogeneity within in vitro nanotoxicological techniques [183]. Due to these difficulties, health and toxicity risk assessments require reliable nanomaterial toxicity and a mechanistic understanding of nanomaterial interactions in physiological systems. One of the strategies that have been introduced to design a toxic-free drug delivery system is the use of biomaterial and chemicals which are non-toxic or that are converted into non-toxic degraded products. Like any other nanodrug delivery system, the biodegradability of materials used in the preparation of nanogels is very crucial to avoid organ accumulation leading to toxicity and other potential side effects. Several studies have shown the use of polysaccharide materials, which are naturally occurring carbohydrate-based polymers, in the preparation of nanogels as biosafe hydrophilic biomaterial [37,184]. In addition, synthetic polymers such as poly(d,l-lactic acid) (PLA), poly(glycolic acid) (PGA) and their copolymer poly(d,l-lactic-co-glycolic acid) (PLGA) have been considered safe and approved by the FDA. Therefore, the nanogels comprising these polymers are expected to retain high viability at high nanogel concentration and to demonstrate minimal toxic potential. Despite the non-toxic nature of these materials of nanogels, a thorough characterization is required before their full potential may be realized. Nanotoxicology, interaction with cells and tissues, including in vivo biodistribution and intracellular trafficking, are some of the challenges that require more investigation [184].

## 10. Conclusions and Future Perspective

Nanogels are new and promising drug-delivery technologies which can play a significant role in addressing clinical issues associated with classic and current therapies or formulations, such as targeted cellular uptake, toxicity-related issues and the development of drug resistance. In this review, we have highlighted different methods of preparation, characterization, properties and biomedical applications of nanogels. The currently reported studies on nanodelivery systems suggest that there is no universal delivery system that can address all the needs of current and future medicine. However, nanogels present some key unique features, including but not limited to stability, biocompatibility, and stimuli responsiveness which can widen their clinical applicability. Nanogel’s superiority as a nanocarrier is highlighted by its capacity to encapsulate and deliver a range of biomolecules of different dimensions. In addition to high encapsulation efficiency, layer-by-layer surface-functionalization can aid in receptor-mediated targeting and subsequent spatiotemporally and sequentially controlled drug release. Nanogels are thought to be the next generation delivery system to improve quality of life based on preclinical data reported in the literature. However, there is still a need for conclusive research demonstrating the potential of nanogels through in vitro and in vivo evaluation. Also, toxicity concerns related to the degraded gel residue, immunogenicity, pharmacokinetic differences between rodent and human models, and regulatory challenges need further exploration. Finally, several researchers have examined the efficacy and safety of nanogel formulations and reports on their long-term accumulation and breakdown characteristics are scarce. However, nanogels as bioactive delivery carriers have a lot of promise for improving medical care efficiency and patient benefit. Therefore, more experimental demonstration is encouraged, from the design to application to better understand the potential of nanogels as the next generation of drug delivery system to fight and eliminate emerging and long standing disease conditions.

## Figures and Tables

**Table 1:** Multi-stimuli-responsive nanogels and their use in biomedical applications.

Nanogel Type	Synthesis Process	Drug	Stimuli Responsiveness	Application	Ref
Paramagnetic iron oxide nanogels composed of paramagnetic iron oxide nanogels composed	Free radical polymerization	Doxorubicin	Novel dual temperature/pH-sensitive	Chemotherapy	[109]
PNIPAM-co-AAc nanogel	Surfactant free emulsion polymerization	β-lipoprotein(β-LP)	Temperature- and pH responsive	Intestine-specific drug delivery	[58]
Poly (N-isopropyl acrylamide-co-acrylic acid) nanogels	Free radical precipitation polymerization	Doxorubicin	Thermo- and pH responsive	Anti-tumor drug delivery	[110]
Ketal derivative, mPEG2000-Isopropylideneglycerol (mPEG-IS, PI) polymer	Self-assembled micellar nanogel	Paclitaxel (PTX)	Dual thermoresponsive and pH-responsive	Cancer therapy	[111]
P(CPT-MAA) prodrug nanogels	Distillation-precipitation polymerization	Camptothecin	pH/redox dual-responsive	Anti-tumor drug delivery	[112]
PAA-based nanogels	Reflux-precipitationpolymerization (RPP)	Cisplatin/Doxorubicin	GSH/pH dual stimuli-responsiveness	Combination Chemotherapy	[113]
P(NIPAM-ss-AA) nanogel	Precipitation polymerization	Doxorubicin	pH/redox dual responsive	Intracellular anticancer drug release	[114]
PEGylated PMAA (PEG-PMAA) nanohydrogels	Facile reflux-precipitation polymerization	Doxorubicin/ Paclitaxel	Redox/pH dual stimuli-responsive	Anti-cancer therapy	[57]
Dendritic polyglycerol (dPG) nanogel	Surfactant-free inverse nanoprecipitation	methotrexate (MTX)	ATP and pH dual-responsive	Anti-cancer therapy	[115]

**Table 2:** Characteristics and general biomedical applications of different types of nanogels in chemotherapy.

Nanogel	Description	Drug Delivered	Application	Pre-Clinical/Clinical Study	Ref
DMMA-modified nanogel	Acidity-activated charge-conversional nanogel as an intelligent vehicle for promoted tumoral-cell uptake and Dox delivery	Dox	Chemotherapy	In vitro cell viability of MDA-MB-435s cells	[75]
polysaccharide-based nanogel	Hyaluronic-Acid-Based pH-Sensitive Nanogels	Dox	Chemotherapy	In vitro cell viability and In vivo mice model tumor volume evaluation	[146]
PEGylated PMAA-based nanogel	Glutathione-sensitive nanogel	Dox	Chemotherapy	In vitro cell viability of HepG2 cells	[147]
HA-based nanogel	Self-targeting hyaluronate (HA) nanogels (^CDDP^HANG/Dox)	Dox and Cisplatin	Chemotherapy	In vitro cell viability of MCF-7/ADR breast cancer cells	[113,148]
pNIPAAm nanogels	Photothermo-Responsive Nanogel	5-fluorouracil	Chemo-photothermal synergistic therapy	In vitro L929 cell mortality	
GO-hybridized pNIPAM nanogels	Photothermally Enhanced Chemotherapy Delivered by Graphene Oxide-based Multi-responsive Nanogels	Dox	Chemo-photothermal synergistic therapy	In vitro cell viability of HeLa cells	[149,150]
Folated PVA-based nanogels	Folated pH degradable PVA nanogels	Docetaxel	Chemo-immunotherapy	In vitro cell viability of 4T1 breast cancer cells	[151]
